# Exploratory Serum Metabolomics Identifies Metabolic Subgroups Across the Gastric Dysplasia-Early Cancer Spectrum

**DOI:** 10.7150/jca.131608

**Published:** 2026-04-23

**Authors:** Sihyun Chae, Hee Sang You, Jae Gyu Kim, Joo-Youn Cho

**Affiliations:** 1 Department of Biomedical Sciences, Seoul National University College of Medicine, Seoul 03080, South Korea.; 2 Department of Clinical Pharmacology and Therapeutics, Seoul National University College of Medicine and Hospital, Seoul 03080, South Korea.; 3 Department of Internal Medicine, Chung-Ang University College of Medicine, Seoul 06974, South Korea.

**Keywords:** Metabolomics, Gastric cancer, Stomach neoplasm, Carcinogenesis, Microbiome

## Abstract

**Background:**

Gastric carcinogenesis involves progressive molecular and metabolic alterations, yet non-invasive biomarkers for early detection and risk stratification remain limited. This study aimed to characterize systemic metabolomic changes across the gastric carcinogenesis spectrum and to investigate potential associations between serum metabolites and gastric microbiome-related pathways.

**Methods:**

In this exploratory study, untargeted serum metabolomics was performed on samples from patients with gastric dysplasia or early gastric cancer (n = 40) and healthy controls (n = 14). Differential metabolite analysis, principal component analysis, and k-means clustering were used to identify metabolic alterations and potential metabolic subgroups. Microbial pathway associations were examined using metabolite origin inference based on gastric-resident taxa reported in prior studies.

**Results:**

Eighteen metabolites were significantly altered in gastric carcinogenesis compared with healthy controls. Six metabolites displayed distinct profiles that suggest two metabolic subgroups, including one subgroup showing a metabolic pattern closer to that of healthy controls, independent of histologic severity. Microbial pathway inference suggested contributions from Pseudomonadota, Actinomycetota, and Bacillota, with ornithine-related metabolites emerging as a key metabolic link previously implicated in dysplasia-to-carcinoma progression. These findings highlight inter-patient heterogeneity and potential metabolic-microbial interactions underlying gastric carcinogenesis.

**Conclusion:**

These findings suggest that serum metabolomic profiling may capture metabolic heterogeneity across the gastric dysplasia-early gastric cancer spectrum and generate hypotheses regarding microbiome-related metabolic alterations. While exploratory in nature, this study provides preliminary evidence supporting the potential of serum metabolites as non-invasive indicators of early gastric carcinogenesis, warranting validation in larger and longitudinal cohorts.

## Introduction

Gastric cancer (GC) is the third leading cause of cancer-related death worldwide, despite being the fifth most common malignancy 1. Despite advancements in diagnostic and therapeutic approaches, GC continues to account for approximately 660,000 deaths annually, with particularly high prevalence observed in East Asia and Eastern Europe. This regional disparity is closely linked to socioeconomic factors, dietary habits, and differences in healthcare infrastructure, which influence the early detection and management of GC 2.

The development of GC is a multifactorial process characterized by a sequence of pathological changes, including chronic and atrophic gastritis and intestinal metaplasia, dysplasia, and, ultimately, adenocarcinoma 3. Genetic predispositions, environmental exposures, and microbial factors are known to influence this multistep progression. Among these, *Helicobacter pylori* plays a pivotal role in the initiation and promotion of gastric carcinogenesis 4. Indeed, the relationship between gastric bacteria and GC has been investigated extensively, with several studies highlighting the impact of microbial diversity on cancer progression 5, 6. Furthermore, recent data have suggested that the gastric microbiome contributes significantly to GC pathogenesis through complex host-microbe interactions 7. This has led to increased interest in using metabolomic approaches to decipher the host-microbiome axis and the role of this axis in GC pathogenesis 8. Notably, while many existing studies focus on the gut microbiome 9-13, this study extends from our previous research on the gastric microbiome 7, which directly interacts with the gastric mucosa and may produce metabolomic signatures more relevant to local tumorigenic processes.

As the need for non-invasive diagnostic and risk-assessment biomarkers grows, metabolomics has emerged as a key tool in biomarker research in systems biology. Recent studies have reported systemic metabolome alterations associated with GC and precancerous lesions, identifying potential diagnostic 14, 15 and predictive biomarkers 16, 17. Recently developed methodologies such as integrative multi-omics or machine-learning algorithms also have been applied for studies about gastric cancer pathophysiology, resulting in metabolic-related signatures of gastric cancer development 18. Additionally, in addition to the well-established role of *H. pylori*, increasing evidence indicates that other gastric microbiota is involved in gastric carcinogenesis 19-22. Gastric mucosal dysbiosis and inflammation have been reported to be linked with alterations in systemic metabolic profiles, particularly amino acid and glutathione pathways, suggesting that serum metabolites may partially reflect gastric microenvironmental changes 6. These observations have motivated recent metabolomic investigations exploring the relationship between gastric microbiota and the pathophysiology of GC 21, 23. While tissue metabolomics has provided valuable insights into local gastric carcinogenesis, serum or plasma metabolome-based studies have increasingly identified systemic metabolic alterations associated with GC 24-26. However, serum metabolomic profiling specifically addressing the dysplasia-early cancer spectrum and potential metabolic subtypes remains relatively less characterized, particularly with respect to potential links to the gastric microbiome.

Although metabolomics is promising for discovering new biomarkers, genomics has been used to categorize the molecular characteristics of GC into four subtypes 27. These subtypes provide the foundational framework for precision oncology and multi-omics approaches, such as proteogenomics, to elucidate GC biology 28. These findings further highlight the need for a deeper understanding of the disease, particularly the potential existence of metabolic subgroups among patients with GC.

Thus, this study performed untargeted serum metabolomics to profile systemic metabolic alterations across the gastric carcinogenesis spectrum and to explore their potential interactions with gastric microbial pathways. Investigating serum metabolic profiles linked to the gastric microbiome offers a promising avenue to uncover novel, non-invasive biomarkers and patient-specific metabolic phenotypes. These integrative approaches may improve our understanding of tumor heterogeneity and support future efforts toward more refined risk stratification and earlier detection in patients with precancerous gastric lesions. Given clinical heterogeneity among patients with gastric dysplasia and early gastric cancer, identifying systemic metabolic fingerprints associated with microbial pathways may complement existing endoscopy-based assessment and provide a framework for future studies on metabolome-informed diagnostic and therapeutic strategies in GC.

## Materials and Methods

### Study design and overview

**Figure 1:**
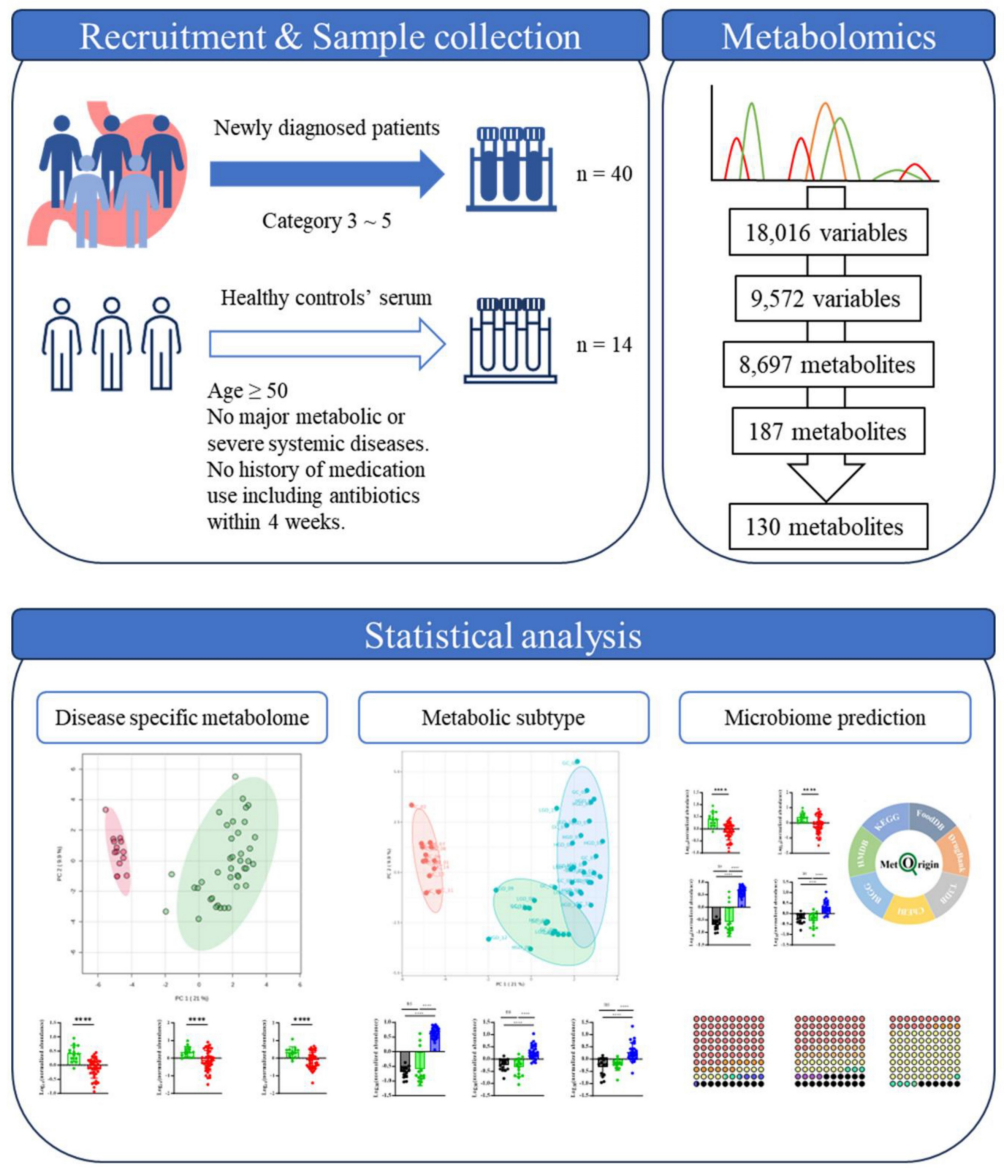
Descriptive workflow of data analysis with selection or exclusion criteria and resulting numbers of metabolites of each step.

This study employed an exploratory serum metabolomics approach to investigate systemic metabolic alterations across the gastric dysplasia-early gastric cancer spectrum. The overall analytical workflow included participant recruitment, serum metabolomic profiling, metabolite feature processing, statistical analysis, and clustering analysis to explore potential metabolic subgroups among patients with gastric carcinogenesis. A schematic overview of the study design and analytical workflow is presented in Figure 1.

### Study participants and inclusion criteria

Participants were recruited at the Chung-Ang University Hospital (Seoul, South Korea) between December 2016 and October 2022. The inclusion criteria for the neoplasm group were newly diagnosed patients with histologically confirmed GC or adenoma. The control samples used for this study were obtained from the Ajou University Hospital (Suwon, South Korea) Human Resource Bank (approval number for sample distribution: AJHB-2024-14). The inclusion criteria for the control group included men and women aged 50 years or older, with no history of cancer, diabetes, obesity, or other major metabolic or severe systemic diseases. Additionally, individuals with no history of medication use, including antibiotics, within 4 weeks prior to sample collection were included in the final selection. The final cohort comprised 54 participants, including 40 patients and 14 controls. This study was approved by the Institutional Review Board of the Chung-Ang University Hospital (IRB number C2016047[1790]) and adhered to the Declaration of Helsinki. All participants provided written informed consent. Gastric neoplasms were diagnosed and categorized using the Vienna Classification System 29. Specifically, category 3 (non-invasive low-grade adenoma/dysplasia) was classified as low-grade dysplasia (LGD), category 4.1 (high-grade adenoma/dysplasia) and 4.2 (non-invasive carcinoma (carcinoma in situ)) were classified as high-grade dysplasia (HGD), and categories 4.3 (suspicion of invasive carcinoma) and 5 (invasive neoplasia) were classified as GC.

### Sample collection and serum preparation

For serum collection, 3 mL of blood was collected from each participant in an SST tube. The tube was then centrifuged (3000× g, 15 min, 4 ℃) immediately after collection, and the serum was extracted. The supernatants (1 mL each) were stored in Eppendorf tubes at -80 °C. All participants were in a fasting state for at least 8 hours prior to blood collection. Blood samples were collected immediately before endoscopic submucosal dissection or surgery, although the interval between initial diagnosis and sample collection varied among participants, ranging from approximately 1 month to several months. Samples were collected between November 13, 2022 and November 27, 2023, and metabolomics analysis was performed in May 2024. For metabolite extraction, 100 µL of 50% high-performance liquid chromatography (HPLC) grade methanol (J.T.Baker^®^ by Avantor, Inc., Radnor, PA, USA) was added to 100 µL of each sample, followed by centrifugation at 14,000 rpm for 5 min. Then, 10 μL of each supernatant was reserved for quality control samples, and the remaining supernatant was transferred into autosampler vials (MicroSolv Technology Corp., Greater Wilmington, NC, USA) and maintained at 4 °C during analysis.

### Metabolomics data acquisition and preprocessing

Metabolomic profiling was performed using an Orbitrap Exploris 120 mass spectrometer (Thermo Fisher Scientific Inc., Waltham, MA, USA) coupled to a Vanquish ultra-HPLC system (Thermo Fisher Scientific Inc.). Metabolite separation was achieved with an ACQUITY UPLC HSS T3 column (100Å, 1.8 µm, 2.1 mm × 100 mm; Waters Corp., Milford, MA, USA) maintained at 40 °C. The mobile phase consisted of A: 0.1% formic acid in methanol and B: 0.1% formic acid in water, with a gradient elution method as follows: 0-0.5 min, 5% solvent B; 0.5-3.5 min, 5% to 95% solvent B; 3.5-9.5 min, 95% solvent B; 9.5-10 min, 95% solvent B; 10-15 min, 95% solvent B; 15-16.5 min, 95% solvent B; 16.5-17.5 min, 95% to 5% solvent B; 17.5-20 min, 5% solvent B. The flow rate was set at 0.35 mL/min for 0-9.5 min, raised to 0.4 mL/min for 9.5-15 min, and returned to 0.35 mL/min for 15-20 min. The sample injection volume was 4 μL. The mass spectrometer was operated in full mass spectrometry (MS) scan mode with a m/z range of 67-1000, a resolution of 120,000, and a spray voltage of 4000 V for positive mode and 3000 V for negative mode. Additional MS2 spectra were obtained from the pooled quality control samples using data-dependent acquisition with a collision energy range of 10-50. The raw data files were converted into mzXML or mgf format using ProteoWizard (ProteoWizard, Palo Alto, CA, USA) 30. Peak picking, alignment, outlier detection, missing value imputation, and normalization were then performed using the TidyMass R package (version 1.0.9) 31, following the recommended quality control procedures implemented in the package. Out of total 18,016 variables (8487 for positive mode and 9529 for negative mode), variables with more than 20% missing values in QC samples and more than 50% missing values in samples are excluded, resulting in total 9572 variables (4356 for positive mode, 5216 for negative mode). These remaining missing values were imputed using k-nearest neighbors algorithm. After initial processing, the resulting features were annotated using public databases from TidyMass and the massDatabase R package (version 1.0.10) 32 and exported for further analysis.

### Statistical analysis

The statistical analysis was performed using MetaboAnalyst 33. A raw p-value cut-off of 0.05 and fold change cut-off of 1.5 were applied for differentially expressed metabolite selection. After statistical selection, each candidate was manually evaluated based on identification (ID) score, MS/MS spectral quality, duplication across features, and potential drug-relatedness.

After selecting metabolite candidates, linear regression models were applied through IBM SPSS (IBM SPSS Statistics for Windows, Version 29.0) to evaluate potential confounding effects of age and sex by comparing the changes in coefficient of each metabolite, with metabolite intensity as the dependent variable and disease status as the independent variable, adjusting for age and sex as covariates.

Unsupervised clustering analysis was performed to explore potential metabolic subgroups among patients with gastric carcinogenesis. K-means clustering was applied to metabolomic features to identify subgroup structures within the patient cohort. The number of clusters (k) was determined based on a combination of clustering evaluation metrics including inspection of within-cluster sum of squares (elbow method) and silhouette width analysis. Because k = 2 primarily separated healthy controls from patients, we focused on k ≥ 3 to identify potential subgroup structure within the patient cohort. The final number of clusters was selected based on the elbow plot, silhouette width, and overall interpretability of the clustering pattern. Based on these criteria, k = 3 provided the most interpretable clustering structure for the dataset. Clustering robustness was assessed by calculating the Adjusted Rand Index (ARI) for the selected k value and, for comparison, neighboring values.

Additional clustering analyses using alternative values of k were also explored to assess robustness. However, higher cluster numbers did not yield stable or biologically interpretable subgroup patterns. As an alternative clustering approach, a Gaussian mixture model was applied using the mclust R package 34 to assess whether the clustering pattern identified by k-means was reproducible.

For pathway-based microbiome analysis, a simple metabolite origin analysis (SMOA) 8 was applied using MetOrigin 2.0 35. Significantly altered metabolites with valid Kyoto Encyclopedia of Genes and Genomes (KEGG) and Human Metabolome Database (HMDB) IDs were used as input data. The results were exported and further visualized with the Alluvium plot R package 36 and GraphPad Prism (version 10.0.2, GraphPad Software, Boston, MA, USA).

## Results

### Clinical characteristics of participants

The clinical characteristics of the study participants are summarized in Table 1. The study included 54 participants, grouped as follows: healthy controls (n = 14), LGD (n = 15), HGD (n = 12), and GC (n = 13). The sex distribution across the healthy controls was balanced, with 50.0% men and 50.0% women. In the LGD group, men represented 60.0%, while women accounted for 40.0%. The HGD group contained more men (75.0%) than women (25.0%). Conversely, the GC group showed a predominance of women (53.8%) over men (46.2%). The healthy controls had a mean age of 55.4 ± 0.4 years, while participants with LGD, HGD, and GC had mean ages of 67.8 ± 2.0, 69.0 ± 1.8, and 68.2 ± 9.1 years, respectively. No participants in the healthy controls, LGD, or HGD groups exhibited lymphovascular invasion. However, one participant in the GC group showed evidence of lymphovascular invasion. No *H. pylori* infections were detected in the healthy controls, LGD, or GC groups; however, one male participant in the HGD group tested positive for *H. pylori*. None of the participants in any group reported using antibiotics or proton pump inhibitors within 4 weeks prior to blood sample collection.

### Patients with gastric carcinogenesis displayed distinct metabolome profiles

**Figure 2:**
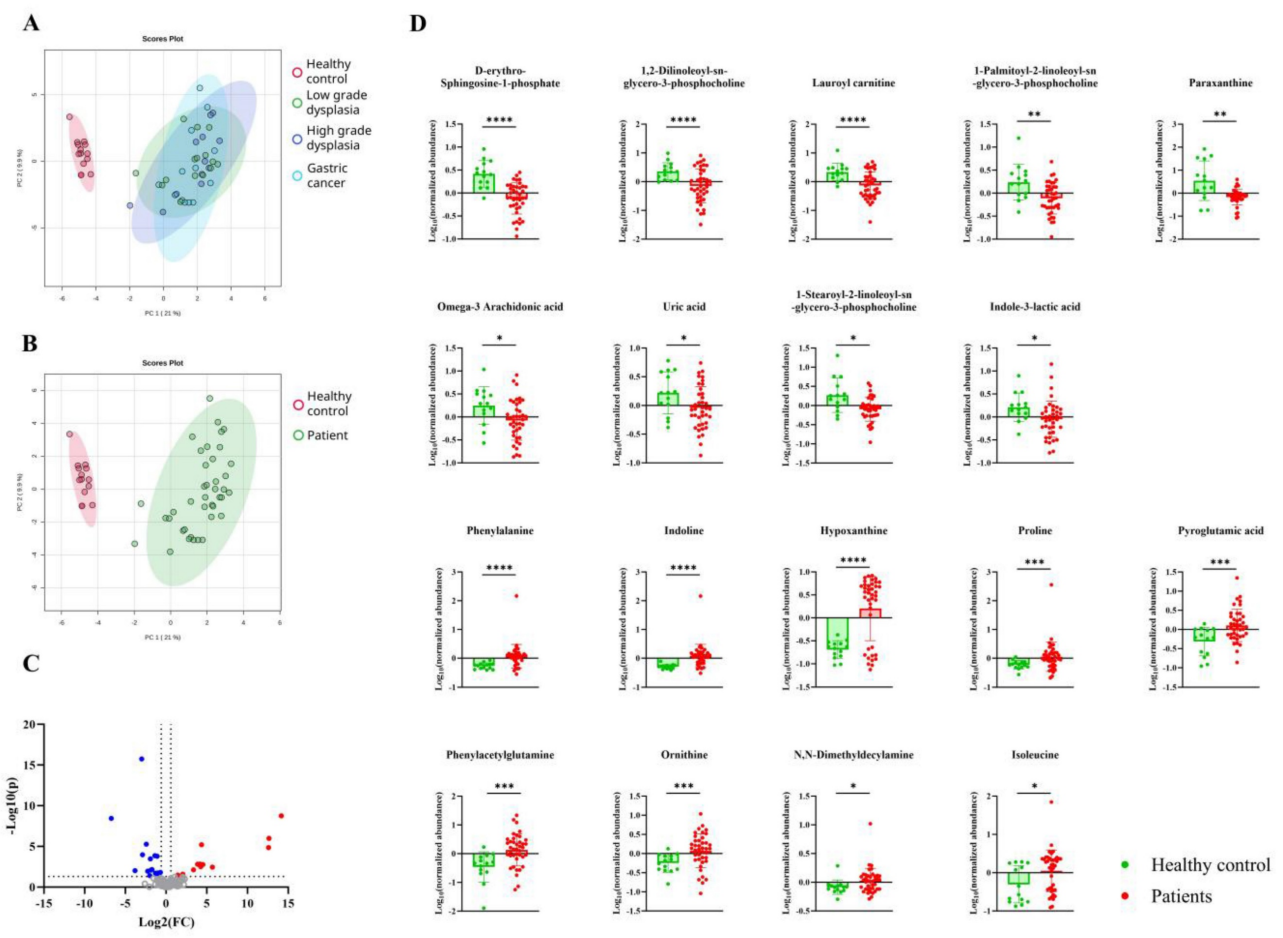
Serum metabolome changes associated with gastric carcinogenesis. **A-B**. Principal component analysis plot showing distinct metabolite composition differences between **(A)** healthy controls (red), patients with low-grade dysplasia (green), high-grade dysplasia (blue), gastric cancer (light blue), **(B)** healthy controls (red) and overall patients (green). The oval areas represent a 95% confidence interval. X and y axis represent two highest variances in the data (Principal component 1 and 2, respectively). **C**. Volcano plot showing differentially expressed metabolites in patients with gastric carcinogenesis compared to healthy controls. **D**. Eighteen metabolites with significant differences (p < 0.05) between patients with gastric carcinogenesis (red) and healthy controls (green).

Serum metabolomics data from patients with gastric carcinogenesis and healthy controls were first analyzed using principal component analysis (PCA). The PCA plot revealed that the systemic metabolomic profiles of patients with gastric carcinogenesis were significantly different from those of the healthy controls (Fig. 2B), without any differences associated with disease severity (Fig. 2A). After validating the annotation and excluding exogenous metabolites, a total of 18 metabolites was selected as being significantly different between the healthy controls and patients with gastric carcinogenesis (Fig. 2C and 2D). Given the age imbalance between healthy controls and patients, we further evaluated potential confounding effects of age and sex using linear regression models. Ten out of eighteen disease-associated metabolites showed coefficient changes over 20% after inclusion of age and/or sex, indicating partial demographic influence. However, the overall disease-associated metabolic pattern and directionality of effects were largely preserved, supporting that the observed systemic metabolomic differences were not solely attributable to age or sex (Supplementary Table S1). Notably, differences were observed between patients and healthy controls, with metabolites such as hypoxanthine showing patterns of intra-group variation, with some patients showing profiles similar to those of healthy controls. However, these variances showed no significant relationship with the disease severity or clinical data of patients, such as drug treatment history. Therefore, post-analysis clustering of the metabolomic profile was performed for greater insight.

### Post-analysis clustering revealed a distinct subgroup among patients

**Figure 3:**
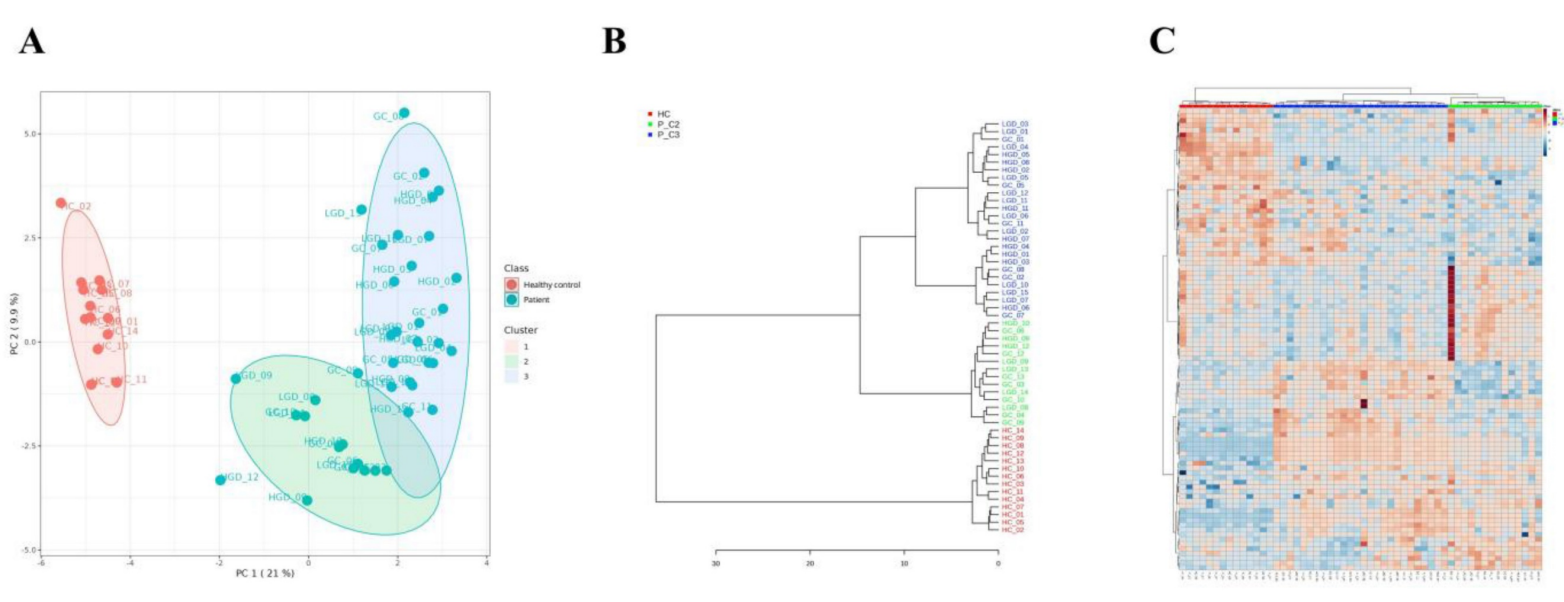
Post-analysis clustering showed two distinct metabolic subgroups of patients. **A**. Three clusters were found based on metabolome data using K-means clustering. Healthy controls (red dots) were clustered as one (red), and patients (blue dots) were divided into two subclusters (blue and green). The oval area represents a 95% confidence interval. **B**. Dendrogram drawn based on the metabolome profile analysis, displaying a clear separation between patients with gastric carcinogenesis, independent of disease status. **C**. Heatmap of 97 metabolites with p < 0.05 based on the ANOVA results. * P_C2, patients with gastric carcinogenesis cluster 2; P_C3, patients with gastric carcinogenesis cluster 3.

Unsupervised k-means clustering of the full dataset identified three clusters overall, consisting of one healthy control cluster and two patient subgroups. The value of k was selected based on elbow method and silhouette analysis, and clustering robustness was evaluated with the adjusted Rand index (ARI). For comparison, Gaussian mixture model was also applied and supported the same overall clustering structure (Supplementary Figure S1). The majority of patients with gastric carcinogenesis (26 patients, 65%) were clustered as cluster 3 (P_C3), highlighted with a blue oval in the PCA plot; the remaining 14 patients (35%) were clustered as cluster 2 (P_C2), highlighted with a green oval in the PCA plot (Fig. 3A). The P_C2 subgroup included patients whose metabolic profiles were similar to that of the healthy controls, contributing to the inter-patient variability observed in the previous analysis (Fig. 2D), independent of disease severity and drug usage history within the preceding 3 months. The clinical and demographic characteristics were also comparable between P_C2 and P_C3 subgroups (Supplementary Table S2). PCA visualization further illustrated a partial trajectory of the P_C2 subgroup toward the healthy control cluster (cluster 1, highlighted with a red oval). These clustering patterns were also observed using a hierarchical clustering dendrogram and heatmap (Fig. 3B and 3C).

### Distinct serum metabolites associated with metabolome-based subgroups in gastric carcinogenesis

**Figure 4:**
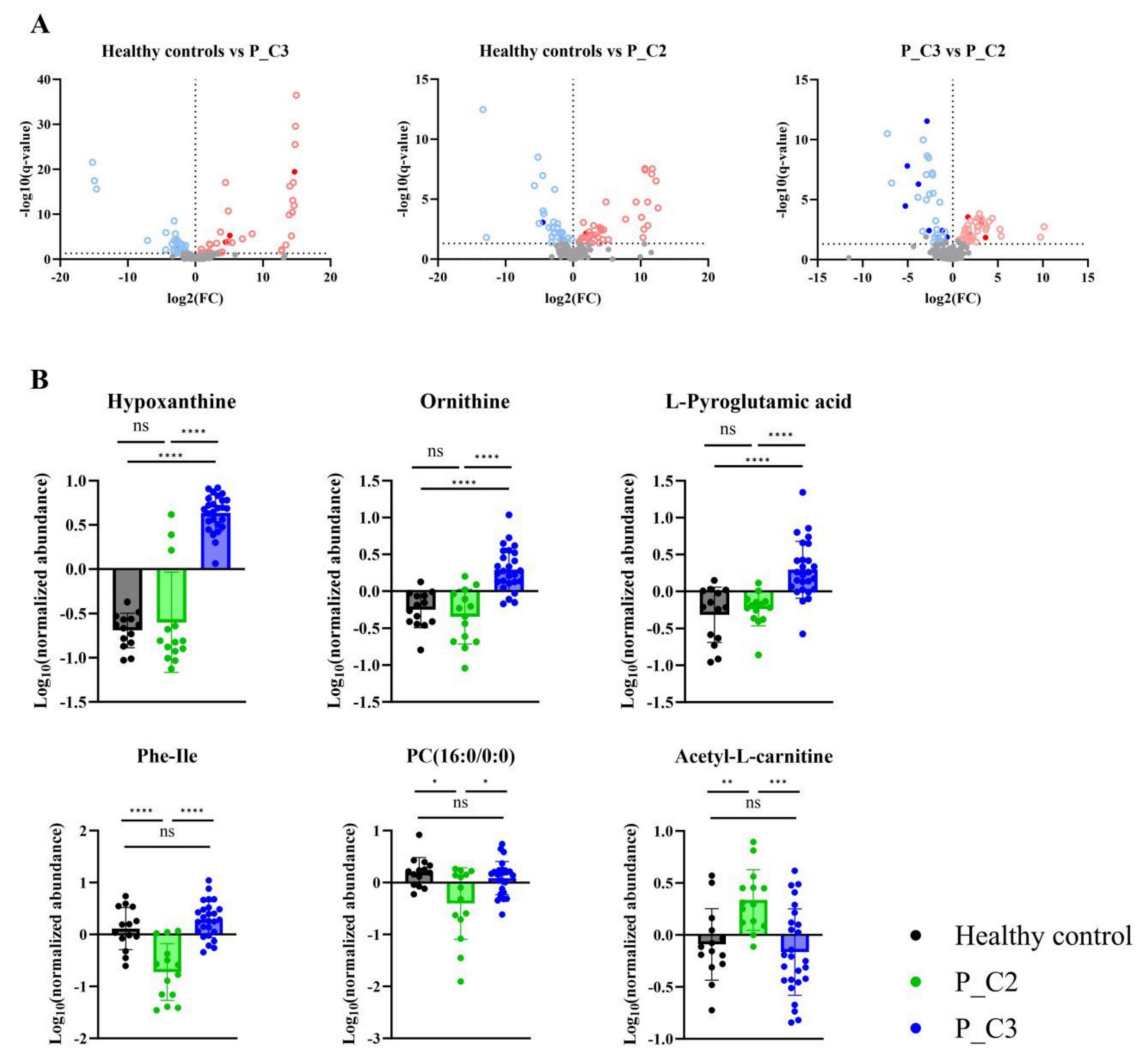
Differentially expressed metabolites in patients with gastric carcinogenesis. **A.** Volcano plots displaying significantly changed metabolites between healthy controls and each patient subgroup (P_C2, P_C3). **B.** Six metabolites showing subgroup specific profiles. * P_C2, patients with gastric carcinogenesis cluster 2; P_C3, patients with gastric carcinogenesis cluster 3.

Differentially expressed metabolites were determined for the healthy controls, P_C2, and P_C3, to further characterize metabolic differences between the identified subgroups (Fig. 4A). As a result, six metabolites that were P_C2- or P_C3-specific were selected (Fig. 4B; Table 2). Although adjustment for age and sex modestly altered the effect sizes of some subgroup-associated metabolites, the overall direction and subgroup-specific patterns of these metabolites were preserved (Supplementary Table S3). In the P_C3 subgroup, hypoxanthine, ornithine, and pyroglutamic acid showed significantly distinguishable profiles from those in P_C2. Conversely, phenylalaninylisoleucine, phosphocholine (16:0/0:0), and acetyl-L-carnitine showed profiles specific to the P_C2 subgroup (Fig. 4B). Among these six identified metabolites, three, namely hypoxanthine, ornithine, and pyroglutamic acid, were also identified as differentially expressed metabolites between the patients with gastric carcinogenesis and healthy controls (Fig. 2C). These findings suggest that heterogeneity in systemic metabolic alterations during gastric carcinogenesis may be partially masked when patients are analyzed as a single group, and that metabolome-based subgrouping may offer additional context for interpreting inter-patient metabolic variability. The biological and clinical significance of these subgroup-associated metabolic patterns warrants further investigation in longitudinal studies.

### Microbial pathway inference indicated gastric microbiome-origin metabolites associated with systemic metabolome changes

**Figure 5:**
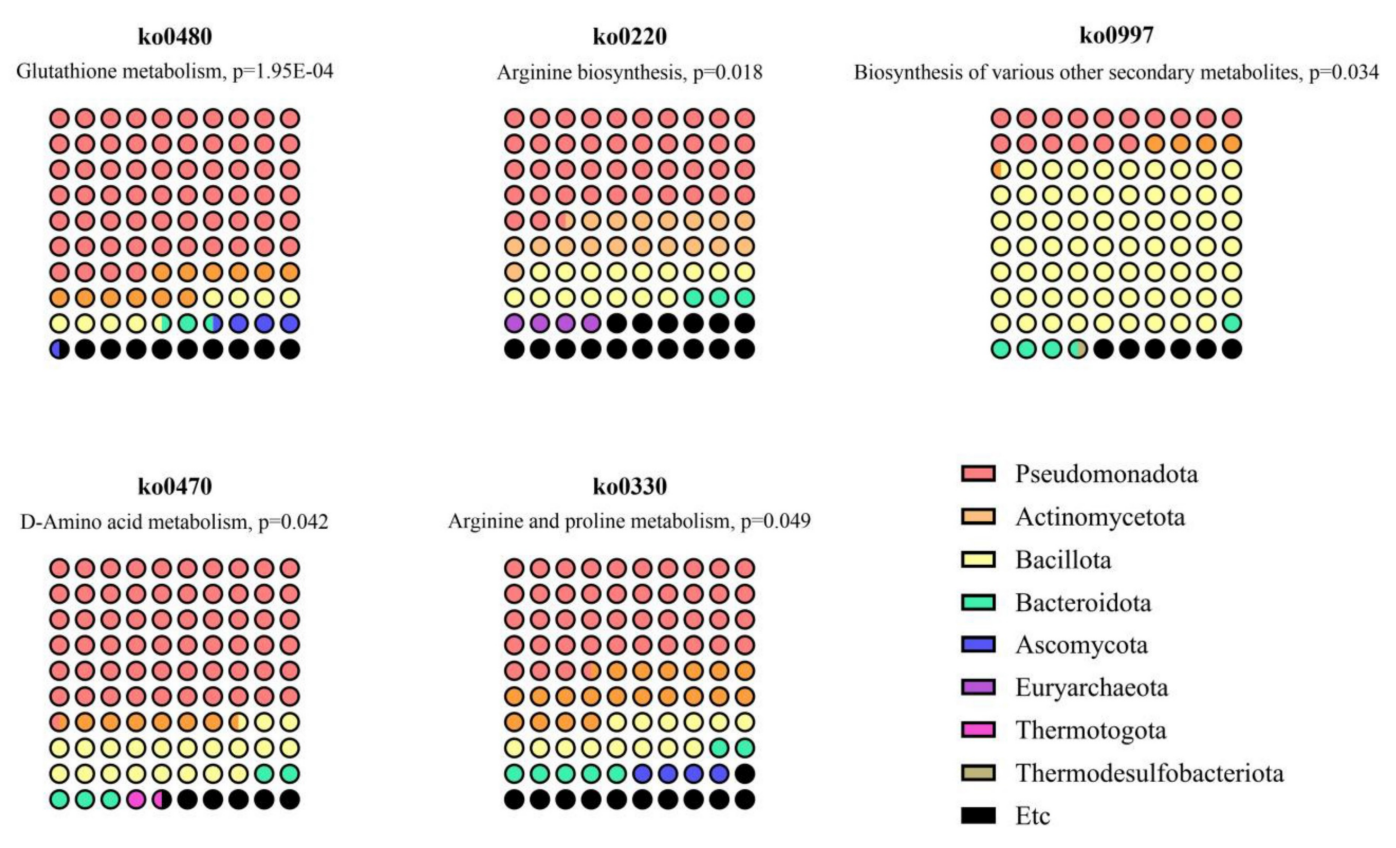
Metabolic pathway-based predictions of gastric microbiota contribution to the differential expression of metabolites in patients with gastric carcinogenesis.

Based on these results, we aimed to explore the possible relationship between the altered systemic metabolome and gastric dysplasia through the gastric microbiome. Therefore, a metabolite origin analysis was conducted to identify specific pathways and bacteria associated with the SMOA metabolomics results 9, 25. First, the KEGG and HMDB databases were used to assign IDs to 18 metabolites that showed significant differences between patients with gastric carcinogenesis and healthy controls. Out of 18 metabolites, 17 metabolites had valid KEGG and HMDB IDs and were used as input data for microbial pathway mapping. As a result, five pathways were predicted putatively to be enriched (p < 0.05) based on 2 metabolites (Fig. 5 and Table 3). Interestingly, the dominant phyla across all five pathways were Pseudomonadota, Actinomycetota, and Bacillota. These pathways included ornithine and pyroglutamic acid, which were differentially expressed in P_C3, the distinctive disease cluster, supporting the possibility of a microbiome-metabolome-related subtype of gastric carcinogenesis at the database level. These pathway-level inferences suggest that altered serum metabolites may be linked to microbial functions previously reported in gastric-resident taxa. However, because the microbiome was not directly profiled in this cohort, these associations should be interpreted as putative and hypothesis-generating rather than causal.

## Discussion

This study aimed to identify the systemic metabolome alterations and any potential associations with gastric microbiota in patients with gastric carcinogenesis. We identified eighteen metabolites that were significantly altered in patients with gastric carcinogenesis compared with healthy controls. Additionally, we identified six metabolites with distinct profiles in patients with gastric carcinogenesis, suggesting the possibility of metabolic subgroups within gastric carcinogenesis. Previous studies have reported metabolomic differences associated with disease severity in gastric carcinogenesis. For example, Huang et al. 16 reported that certain plasma metabolite clusters shift progressively with GC progression, and Choi et al. 21 recently identified distinct metabolomic signatures associated with precancerous lesions and GC. However, our findings depart from these prior reports in a key respect: the identification of a metabolic subgroup (P_C2) with a metabolic profile closer to that of healthy controls, independent of disease severity. While previous studies suggest a linear progression of metabolomic changes, our results indicate heterogeneity among patients, with a subgroup showing metabolic profiles similar to those of healthy individuals. Given the age imbalance in our cohort, we evaluated the potential confounding effects of age and sex on disease-associated metabolites and subgroup assignment. Baseline clinical and demographic characteristics were comparable between the two subgroups, and the overall directionality of disease-associated metabolites and subgroup-specific metabolites was preserved, although adjustment for age and/or sex led to notable effect size changes for a subset of metabolites. This subgroup may reflect inter-patient metabolic heterogeneity in gastric carcinogenesis not captured by histologic staging alone; however, its biological significance remains unclear. The underlying mechanisms for this subgroup remain unclear and require validation in larger longitudinal cohorts but could be influenced by individual differences in microbiome composition, metabolic adaptation, or genetic factors. These findings highlight distinct systemic metabolic alterations accompanying gastric carcinogenesis and reveal metabolic phenotypes that may capture inter-patient heterogeneity.

Furthermore, these findings raise the possibility that gastric microbiome-related pathways may be linked to systemic metabolomic shifts observed in gastric carcinogenesis. While previous studies have predominantly assessed the fecal or intestinal microbiota 7, 9-13, 20, 37-39, our approach focuses on microbial pathways associated with gastric-resident taxa, aligning more closely with the local tumor microenvironment. Because direct gastric microbiome profiling was not available, the microbial pathway associations should be interpreted as putative rather than causal. In addition, we identified Pseudomonadota, Actinomycetota, and Bacillota as the dominant phyla contributing to altered metabolic pathways. Notably, ornithine metabolism, previously reported as being microbiome-associated 40-42 and as key processes in dysplasia-to-carcinoma progression 16, 21, emerged as a key link between the metabolome and microbiota in GC. Altered ornithine availability may contribute to increased polyamine synthesis, oxidative stress, and proliferative signaling in gastric mucosa, providing a biological rationale for its association with early carcinogenic processes.

These findings may provide a conceptual framework for future biomarkers and stratification research in patients with GC. Current non-invasive biomarkers include *H. pylori* serology and pepsinogen levels, which offer limited insight into patient heterogeneity. Studies on GC subgrouping have identified emerging biomarker candidates, such as circulating microribonucleic acids (microRNAs) 43-45, proteins 46, and metabolites 45, which could help address this gap. By identifying metabolic subgroups in patients with GC, our study underscores the potential role of metabolic profiling to refine GC diagnostics.

Despite the strengths presented in this study, several limitations exist. First, the relatively small cohort size (40 patients) limits statistical power. Especially for the clustering analysis, even though the evaluation metrics suggested that k = 3 provided the most interpretable clustering structure in our cohort, larger cohorts will be required to confirm the stability and biological significance of the identified metabolic subgroup. Second, the use of biobank-derived controls rather than prospectively recruited healthy participants may have introduced bias. Additionally, given the younger age distribution of controls, residual confounding cannot be fully excluded despite regression adjustment. Third, this study is cross-sectional, precluding causal inferences about the relationship between microbiome-associated metabolomic changes and disease progression. Hence, longitudinal studies are required to track metabolic changes over time. Fourth, even with support from previously reported results, the lack of direct microbiome analysis in the same participants also requires further validation. Finally, the absence of an independent validation cohort limits the power of our findings, particularly regarding metabolic subgrouping and microbiota-metabolome interactions. Future studies to validate these findings in larger, independent cohorts incorporating paired gastric microbiome and serum metabolome data may enhance the significance and clinical relevance of the findings presented in this study. Nevertheless, these findings provide preliminary but meaningful evidence supporting the utility of systemic metabolomics as a non-invasive tool in gastric carcinogenesis research.

In conclusion, our study provides insights into systemic metabolomic alterations in patients with gastric carcinogenesis and their associated microbial pathways. We identified two metabolic subgroups among patients with gastric carcinogenesis, independent of disease severity, highlighting the heterogeneity of GC-related metabolism. Furthermore, the putative prediction of Pseudomonadota, Actinomycetota, and Bacillota as key microbial contributors suggests a potential link between gastric microbiome dysbiosis and systemic metabolic changes. While further validation is necessary, these findings suggest that metabolomic profiling may support future studies of non-invasive biomarkers, patient stratification, and serum metabolomic heterogeneity, including microbiome-related metabolic signatures, across the gastric carcinogenesis spectrum.

## Supplementary Material

Supplementary figure and tables.

## Figures and Tables

**Table 1:** Basic demographics of study participants.

Variables		Healthy controls	LGD	HGD	GC
Sex	Male	7 (50.0%)	9 (60.0%)	9 (75.0%)	6 (46.2%)
	Female	7 (50.0%)	6 (40.0%)	3 (25.0%)	7 (53.8%)
	Total	14	15	12	13
Age (years)	Male	54.0 ± 0.4	67.3 ± 3.5	67.7 ± 4.0	66.2 ± 3.7
	Female	56.9 ± 0.1	68.5 ± 1.3	73.0 ± 4.0	69.9 ± 5.1
	Total	55.4 ± 0.4	67.8 ± 2.0	69.0 ± 1.8	68.2 ± 9.1
Lymphovascular invasion	Male	0	0	0	0
	Female	0	0	0	1 (14.3%)
	Total	0	0	0	1 (7.7%)
*Helicobacter pylori*	Male	0	0	1 (11.1%)	0
	Female	0	0	0	0
	Total	0	0	1 (8.3%)	0

LGD, Low-grade dysplasia; HGD, High-grade dysplasia; GC, Gastric cancer.

**Table 2:** Metabolites with significant differences between the two subclusters of patients with gastric carcinogenesis.

Specificity	Metabolites	Fold changes	q-values	Identification scores	Identification level
P_C3-specific	Hypoxanthine	0.13721	5.1646E-10	0.846920366	Level 2
	Ornithine	0.070988	8.4375E-06	0.825485567	
	L-pyroglutamic acid	0.026251	0.0003688	0.804260172	
P_C2-specific	Phenylalaninylisoleucine	0.03039	4.7121E-07	0.870166412	
	Acetyl-L-carnitine	3.213	0.0027411	0.804396637	
	Phosphocholine (16:0/0:0)	0.44995	0.016775	0.818053713	

P_C2, patients with gastric carcinogenesis cluster 2; P_C3, patients with gastric carcinogenesis cluster 3.

**Table 3:** Microbiome pathways predicted to be related to metabolome changes in patients with gastric carcinogenesis.

KEGG Orthology	Pathway name	Match status	p-value	Metabolites	Dominant phylum
ko00480	Glutathione metabolism	2 in 32	1.95E-04	Ornithine L-pyroglutamic acid	Pseudomonadota; Actinomycetota; Bacillota
ko00220	Arginine biosynthesis	1 in 23	0.018	Ornithine	Pseudomonadota; Actinomycetota; Bacillota
ko00997	Biosynthesis of various other secondary metabolites	1 in 45	0.034	Ornithine	Bacillota; Pseudomonadota
ko00470	D-amino acid metabolism	1 in 56	0.042	Ornithine	Pseudomonadota; Bacillota; Actinomycetota
ko00330	Arginine and proline metabolism	1 in 65	0.049	Ornithine	Pseudomonadota; Actinomycetota; Bacillota

## Data Availability

The data of this study have been submitted to Korea BioData Station (kbds.re.kr) and Korea MetAbolomics data repository (KMAP). The data will be available upon request and with the permission of the authors.
