# Supplementary material for: Exploratory Serum Metabolomics Identifies Metabolic Subgroups Across the Gastric Dysplasia-Early Cancer Spectrum
Source: J Cancer. 2026 Apr 23;17(5):968–78. doi: 10.7150/jca.131608 (PMC13189843; doi:10.7150/jca.131608)
Supplement: Supplementary file 1 — Supplementary figure and tables. [file jcav17p0968s1.pdf]

Supplementary materials

Supplementary Table S1. Assessment of age- and sex-related confounding effects on disease-associated serum metabolites using linear regression models.

Supplementary Table S2. Clinical Characteristics of metabolic subgroups and healthy controls.

Supplementary Table S3. Assessment of age- and sex-related confounding effects on cluster-specific serum metabolites using linear regression models.

Supplementary Figure S1. The results of within-cluster-sum-squared analysis (Elbow analysis), Silhouette analysis, Robustness analysis with adjusted Rand index (ARI) and Gaussian mixture model for k value.

Supplementary Table S1. Assessment of age- and sex-related confounding effects on disease-associated serum metabolites using linear regression models (Continues).

Specificity	Metabolite	Metabolites	Metabolites + Age		Metabolites + Sex	
		Standardized coefficient	Standardized coefficient	Change (%)	Standardized coefficient	Change (%)
Disease specific metabolites	D-erythro-Sphingosine-1-phosphate	-0.477	-0.415	12.88	-0.475	0.29
	1,2-Dilinoleoyl-sn-glycero-3-phosphocholine	-0.253	-0.311	23.05	-0.235	7.05
	Lauroyl carnitine	-0.143	-0.165	15.83	-0.151	5.64
	1-Palmitoyl-2-linoleoyl-sn-glycero-3-phosphocholine	0.046	0.030	34.67	0.072	57.46
	Paraxanthine	-0.083	-0.074	11.80	-0.080	4.24
	Omega-3 Arachidonic acid	0.006	-0.053	961.91	0.014	125.52
	Uric acid	-0.151	-0.166	10.26	-0.150	0.20
	1-Stearoyl-2-linoleoyl-sn-glycero-3-phosphocholine	0.017	0.088	418.58	0.007	61.10
	Indole-3-lactic acid	0.034	0.052	50.76	0.007	78.98
	Phenylalanine	-2.201	-2.102	4.47	-2.116	3.87

Change (%) = |Adjusted coefficient - Unadjusted coefficient| / |Unadjusted coefficient | × 100.

Supplementary Table S1. Assessment of age- and sex-related confounding effects on disease-associated serum metabolites using linear regression models (Continues).

Specificity	Metabolite	Metabolites	Metabolites + Age		Metabolites + Sex	
		Standardized coefficient	Standardized coefficient	Change (%)	Standardized coefficient	Change (%)
Disease specific metabolites	Hypoxanthine	0.499	0.276	44.67	0.468	6.20
	Ornithine	-0.087	-0.082	5.68	-0.035	59.79
	L-Pyroglutamic acid	0.145	0.221	52.12	0.158	9.02
	Phe-Ile	-0.630	-0.487	22.69	-0.644	2.13
	PC(16:0/0:0)	-0.209	-0.107	48.63	-0.212	1.74
	Acetyl-L-Carnitine	0.234	0.247	5.74	0.235	0.45
	Age	N/A	0.386	N/A	N/A	N/A
	Sex	N/A	N/A	N/A	0.073	N/A

Change (%) = |Adjusted coefficient - Unadjusted coefficient| / |Unadjusted coefficient | × 100.

Supplementary Table S2. Clinical Characteristics of metabolic subgroups and healthy controls.

Variables		Healthy controls	P_C2	P_C3
Sex	Male	7 (50.0%)	7 (50.0%)	17 (65.0%)
	Female	7 (50.0%)	7 (50.0%)	9 (35.0%)
	Total	14	14	26
Age (years)	Male	54.0 ± 0.4	72.5 ± 11.0	64.0 ± 9.1
	Female	56.9 ± 0.1	69.5 ± 9.1	69.1 ± 9.8
	Total	55.4 ± 0.4	71.0 ± 9.8	65.7 ± 9.5
Lymphovascular invasion	Male	0	0	0
	Female	0	0	1 (11.1%)
	Total	0	0	1 (3.8%)
Helicobacter pylori	Male	0	0	1 (5.9%)
	Female	0	0	0
	Total	0	0	1 (3.8%)

Statistical comparisons: P_C2 vs P_C3 – Age – Male: p=0.0524, Female: p=0.7402, Total: p=0.0974, P_C2 vs P_C3 – Sex: p=0.5001. No significant differences observed.

Supplementary Table S3. Assessment of age- and sex-related confounding effects on cluster-specific serum metabolites using linear regression models.

Specificity	Metabolite	Metabolites	Metabolites + Age		Metabolites + Sex	
		Standardized coefficient	Standardized coefficient	Change (%)	Standardized coefficient	Change (%)
Cluster specific metabolites	Hypoxanthine	0.499	0.276	44.67	0.468	6.20
	Ornithine	-0.087	-0.082	5.68	-0.035	59.79
	L-Pyroglutamic acid	0.145	0.221	52.12	0.158	9.02
	Phe-Ile	-0.630	-0.487	22.69	-0.644	2.13
	PC(16:0/0:0)	-0.209	-0.107	48.63	-0.212	1.74
	Acetyl-L-Carnitine	0.234	0.247	5.74	0.235	0.45
	Age	N/A	0.386	N/A	N/A	N/A
	Sex	N/A	N/A	N/A	0.073	N/A

Change (%) = |Adjusted coefficient - Unadjusted coefficient| / |Unadjusted coefficient | × 100.

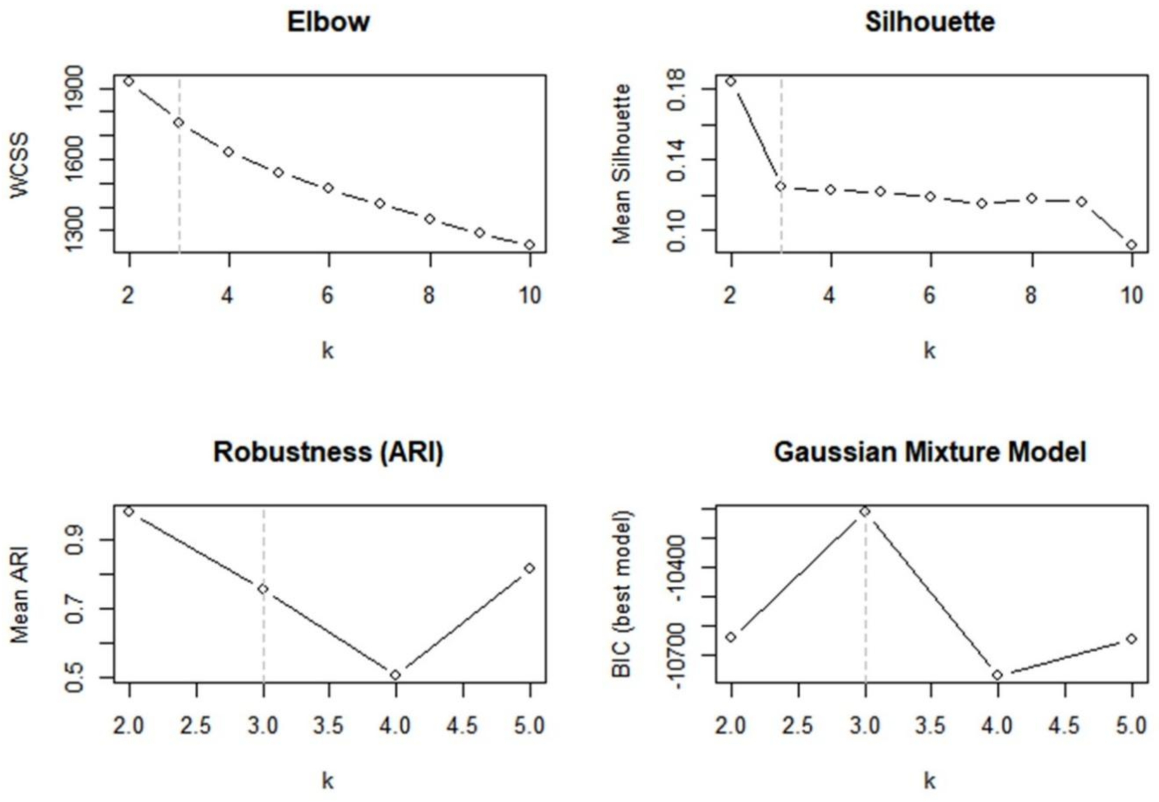

Supplementary Figure S1. The results of within-cluster-sum-squared analysis (Elbow analysis), Silhouette analysis, Robustness analysis with adjusted Rand index (ARI) and Gaussian mixture model for k value.
